# The genetic architecture of plasma kynurenine includes cardiometabolic disease mechanisms associated with the *SH2B3* gene

**DOI:** 10.1038/s41598-021-95154-9

**Published:** 2021-08-02

**Authors:** Minoo Bagheri, Chuan Wang, Mingjian Shi, Ali Manouchehri, Katherine T. Murray, Matthew B. Murphy, Christian M. Shaffer, Kritika Singh, Lea K. Davis, Gail P. Jarvik, Ian B. Stanaway, Scott Hebbring, Muredach P. Reilly, Robert E. Gerszten, Thomas J. Wang, Jonathan D. Mosley, Jane F. Ferguson

**Affiliations:** 1 Division of Cardiovascular Medicine, Department of Medicine, Vanderbilt University Medical Center, 2220 Pierce Ave, PRB 354B, Nashville, TN 37232 USA; 2 Department of Biomedical Informatics, Vanderbilt University Medical Center, Nashville, TN USA; 3 Division of Clinical Pharmacology, Department of Medicine, Vanderbilt University Medical Center, Nashville, TN USA; 4 Division of Genetic Medicine, Department of Medicine, Vanderbilt University Medical Center, Nashville, TN USA; 5 Departments of Medicine (Medical Genetics) and Genome Sciences, University of Washington, Seattle, WA USA; 6 Division of Nephrology, School of Medicine, Harborview Medical Center Kidney Research Institute, University of Washington, Seattle, WA USA; 7 Center for Precision Medicine Research, Marshfield Clinic Research Institute, Marshfield, WI USA; 8 Irving Institute for Clinical and Translational Research and Division of Cardiology, Columbia University Medical Center, New York, NY USA; 9 Division of Cardiovascular Medicine, Beth Israel Deaconess Medical Center, Boston, MA USA; 10 Department of Internal Medicine, University of Texas Southwestern Medical Center, Dallas, USA

**Keywords:** Prognostic markers, Cardiovascular diseases, Disease genetics, Gene regulation

## Abstract

Inflammation increases the risk of cardiometabolic disease. Delineating specific inflammatory pathways and biomarkers of their activity could identify the mechanistic underpinnings of the increased risk. Plasma levels of kynurenine, a metabolite involved in inflammation, associates with cardiometabolic disease risk. We used genetic approaches to identify inflammatory mechanisms associated with kynurenine variability and their relationship to cardiometabolic disease. We identified single-nucleotide polymorphisms (SNPs) previously associated with plasma kynurenine, including a missense-variant (rs3184504) in the inflammatory gene *SH2B3/LNK*. We examined the association between rs3184504 and plasma kynurenine in independent human samples, and measured kynurenine levels in *SH2B3*-knock-out mice and during human LPS-evoked endotoxemia. We conducted phenome scanning to identify clinical phenotypes associated with each kynurenine-related SNP and with a kynurenine polygenic score using the UK-Biobank (n = 456,422), BioVU (n = 62,303), and Electronic Medical Records and Genetics (n = 32,324) databases. The *SH2B3* missense variant associated with plasma kynurenine levels and *SH2B3*^−/−^ mice had significant tissue-specific differences in kynurenine levels.LPS, an acute inflammatory stimulus, increased plasma kynurenine in humans. Mendelian randomization showed increased waist-circumference, a marker of central obesity, associated with increased kynurenine, and increased kynurenine associated with C-reactive protein (CRP). We found 30 diagnoses associated (FDR q < 0.05) with the *SH2B3* variant, but not with SNPs mapping to genes known to regulate tryptophan-kynurenine metabolism. Plasma kynurenine may be a biomarker of acute and chronic inflammation involving the *SH2B3* pathways. Its regulation lies upstream of CRP, suggesting that kynurenine may be a biomarker of one inflammatory mechanism contributing to increased cardiometabolic disease risk.

## Introduction

Epidemiological studies have identified numerous biomarkers associated with clinical disease^1^. However, the etiological relationship between the biomarker and disease, and the molecular mechanisms contributing to their association often cannot be determined by traditional association approaches. This knowledge gap limits the opportunities to identify clinical applications for a putative biomarker. Genetic approaches, which link molecular mechanisms with disease outcomes, can ascertain for possible causal relationships between a biomarker and a disease, and can identify the discrete disease-associated mechanisms captured by the biomarker^2^. Here, we leverage such genetic approaches to more fully characterize the plasma metabolite kynurenine.

Kynurenine is a modulatory biomolecule synthesized from the essential dietary amino acid tryptophan, in part, by the inducible enzyme indoleamine 2,3-dioxygenase (IDO)^3^. Plasma kynurenine has been associated with a range of phenotypes including cardiovascular diseases (CVD) [heart disease, atherosclerosis, and endothelial dysfunction], hypertension, diabetes, obesity and neuropsychiatric disorders^3,4^. Animal and cell models suggest that kynurenine pathway metabolites are linked to disease through modulation of inflammation^5,6^. Therapies that decrease inflammation lower levels of non-specific inflammatory biomarkers such as c-reactive protein (CRP)^7^, reduce cardiovascular disease risk^8^ and improve cardiometabolic indices including levels of glucose and insulin which would subsequently lead to disease reduction^9^. However, findings relating the kynurenine pathway to inflammation and cardiometabolic disease are inconsistent, and the mechanistic relationship between plasma kynurenine levels and cardiometabolic disease risk is not well-defined.

We hypothesized that characterizing the genetic determinants underlying plasma kynurenine levels would define its role in health and disease risk. We used single nucleotide polymorphisms (SNPs) associated with plasma kynurenine levels as instrumental variables to determine how genetic regulation of kynurenine contributes to disease risk. We leveraged data from observational and interventional human studies and mouse models and showed that plasma kynurenine is a biomarker associated with multiple cellular mechanisms, and that only one of these mechanisms appears to associate with clinical disease.

## Results

### SNPs in four independent genomic regions associate with plasma kynurenine

Of all SNPs reaching genome-wide significance (*p* < 5 × 10^−8^), we identified six lead SNPs associated with plasma kynurenine in the Cooperative Health Research in the Region of Augsburg (KORA) and TwinsUK GWAS meta-analysis at (Table 1). Based on Linkage disequilibrium (LD) and genomic location, we concluded that these 6 lead SNPs represent 4 independent loci. Three SNPs are cis-expression quantitative trait loci (eQTLs) for genes involved in tryptophan/kynurenine metabolism: the *IDO* 1/2 genes (rs10085935, Chr8), which are the rate limiting enzymes in the kynurenine extrahepatic pathway^3,10^; and *SLC7A5* (rs750950 and rs8051149, Chr16), which participates in tryptophan transport across the cell membrane^11^. Two SNPs mapped to a region on Chr 12. The strongest association was with rs3184504, a missense variant in a known inflammatory gene, *SH2B3*, and has been associated with multiple phenotypes including myocardial infarction and hypertension^12^. The final SNP (rs16924894, Chr10) is an intergenic variant located near the *KIAA1217* and *ARHGAP21* genes. Tryptophan 2,3-dioxygenase, encoded by the TDO2 gene, is another key regulatory enzyme in the kynurenine pathway^13^. We assessed the SNPs located in or near the TDO2 genes and none were significantly associated with plasma kynurenine levels. Of 6 downstream metabolites (3-hydroxy anthranilic acid (3HAA), and quinolinic acid (QA), NAM (nicotinamide), NA (nicotinic acid), KA (kynurenic acid), HK (hydroxykynurenine)) in the kynurenine pathway, GWAS data of QA and NAM levels^14,15^ showed no significant associations with the *SH2B3* rs3184504 variant. (Supplementary Table 1).

**Table 1:** Annotations for top GWAS loci associated with circulating kynurenine levels derived from the KORA-TwinsUK meta-analysis GWAS summary statistics.

SNP	Chromosome	Position	Reference allele	Alternative allele	Reference allele frequency	Beta	SE	P value	Closest gene (s)	Genomic location	eQTL (s)^a^	Possible relationship to kynurenine
rs10085935	8	39,806,267	T	C	0.37	− 0.010	0.002	3.326E−09	IDO2	intronic	IDO1	✓
rs16924894	10	24,845,525	A	T	0.02	0.081	0.015	2.329E−08	KIAA1217 (dist = 8748), ARHGAP21 (dist = 27,013)	intergenic	No significant eQTLs were found	
rs3184504	12	111,884,608	T	C	0.48	0.015	0.002	6.046E−18	SH2B3	exonic-Missense (R262W)	ALDH2, LINC01405, TMEM116, ADAM1B, MAPKAPK5-AS1	✓
rs11066320	12	112,906,415	A	G	0.43	0.012	0.002	3.101E−11	PTPN11	intronic	ALDH2, ADAM1B, MAPKAPK5, NAA25, BRAP	
rs750950	16	87,873,247	A	C	0.64	0.014	0.002	2.342E−16	SLC7A5	intronic	SLC7A5	✓
rs8051149	16	87,878,822	A	G	0.21	0.026	0.003	9.073E−26	SLC7A5	intronic	SLC7A5, RP4-536B24.2	✓

Data was obtained from http://metabolomics.helmholtz-muenchen.de/gwas/. Four shading rows indicate SNPs belonging to 4 independent regions.

*SNP* single nucleotide polymorphism, *SE* standard error.

^a^Data was obtained from https://gtexportal.org/home/

### *SH2B3* and rs3184504 associate with plasma kynurenine

**Figure 1:**
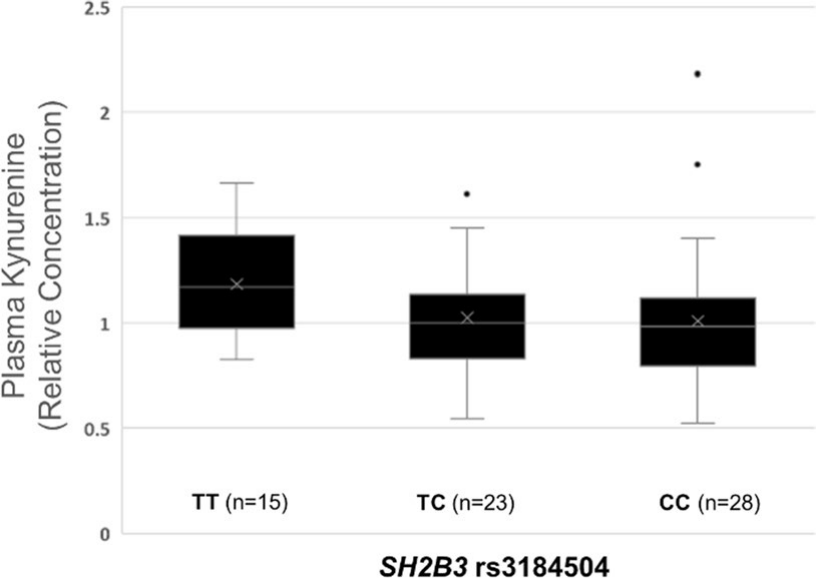
The association of *SH2B3* rs3184504 genotype with plasma kynurenine was confirmed in a sample of 66 healthy men and women. This figure was created in IBM SPSS Statistics 27.

To confirm the *SH2B3* rs3184504 SNP association with kynurenine levels, we examined the genetic association with plasma kynurenine using data from the ABO Glycoproteomics in Platelets and Endothelial Cells (ABO) Study (N = 66 young healthy volunteers). The pattern of association was consistent with a recessive effect (Fig. 1), with a significant association between homozygosity for the T allele and increased kynurenine (β = 0.17, *p* = 0.04, recessive genetic model).

**Figure 2:**
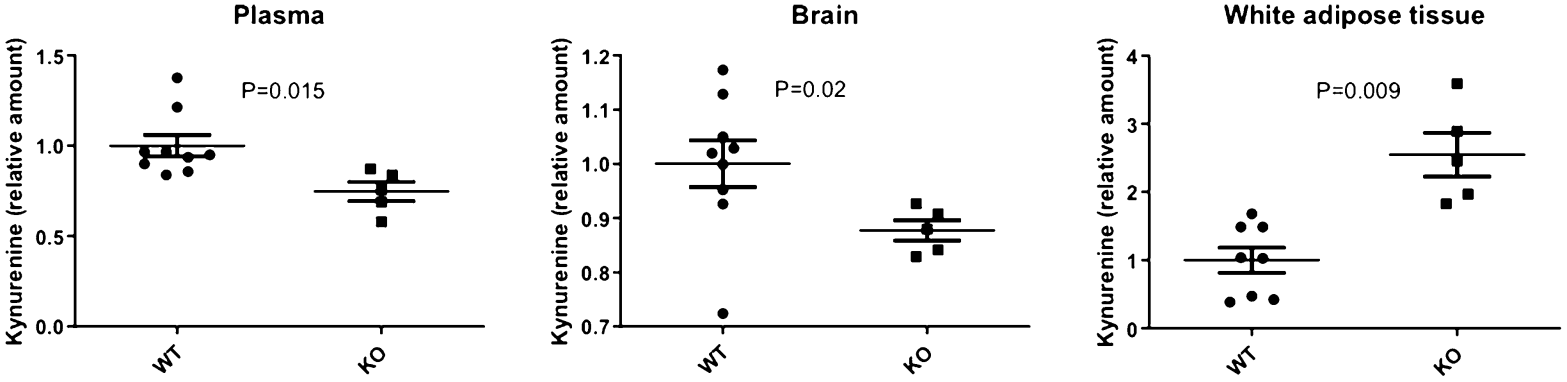
Kynurenine levels in selected tissues in wild type (WT, N = 8) and *SH2B3* knockout (KO, N = 5) mice. Kynurenine levels were measured by ELISA assay. Values are expressed as relative concentrations, normalized to WT. Differences between WT and KO animals were analyzed by unpaired t test. This figure was created in GraphPad Prism v.9.1.1.

We further characterized regulation of kynurenine by *SH2B3* by measuring kynurenine levels in an *Sh2b3*^*−*/*−*^ mouse, as compared to wild-type controls. The most marked differences in kynurenine levels were observed in white adipose tissue where there were more than 2.5-fold (*p* = 0.009) higher kynurenine levels in *Sh2b3*^−/−^ compared to wild type mice (Fig. 2). In contrast, significantly lower levels of kynurenine were observed in plasma (≈ 0.25-fold, *p* = 0.02) and brain (≈ 0.13-fold, *p* = 0.02) of *Sh2b3*^−/−^ mice. There was no difference in kynurenine levels in the spleen and modestly lower, but non-significant, levels in the liver in KO mice compared to controls. (Supplementary Fig. 1). These data suggest that *SH2B3* is a regulator of kynurenine metabolism, but that the effects on the pathway may differ by tissue.

### Plasma Kynurenine and inflammation

**Figure 3:**
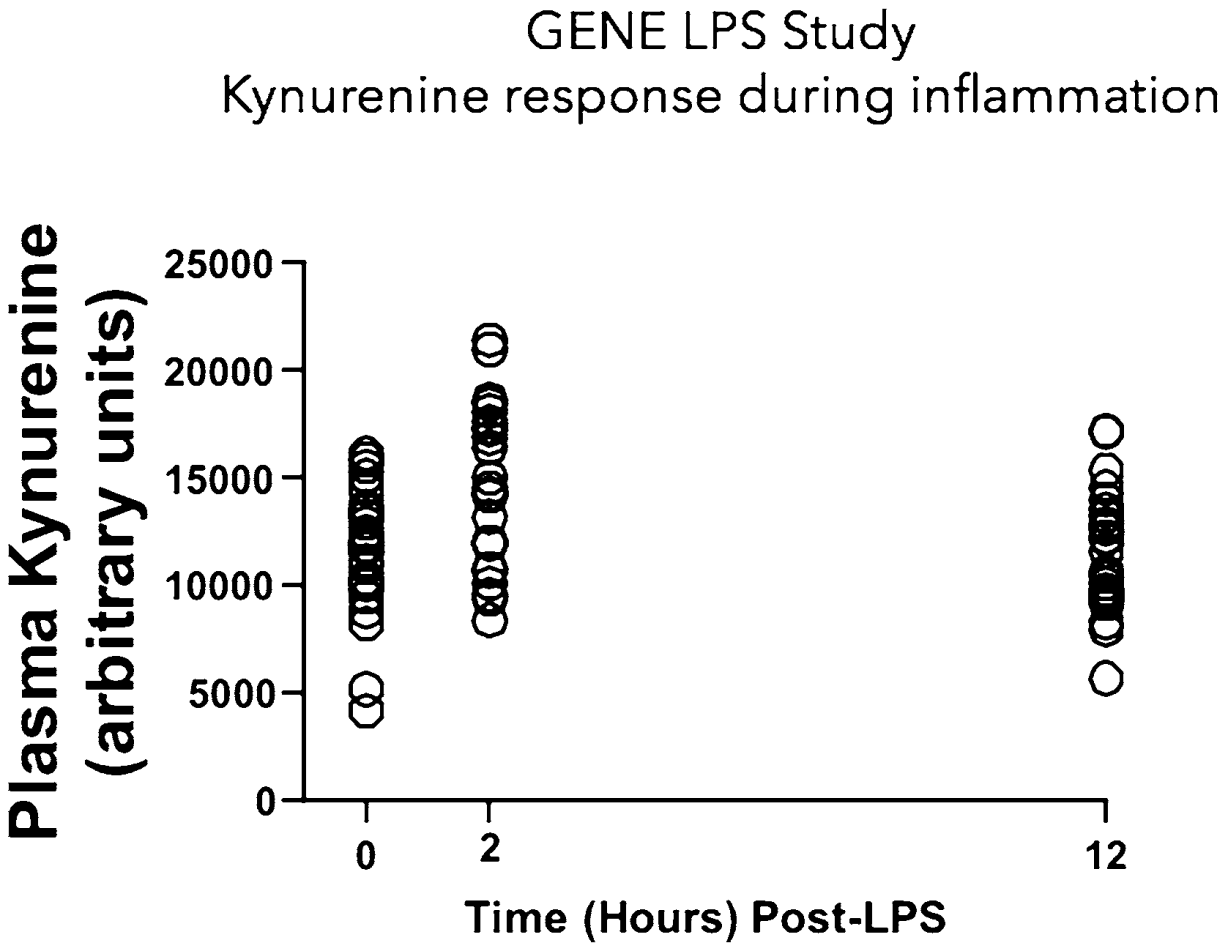
Plasma Kynurenine response to evoked endotoxemia (LPS, 1 ng/kg) in healthy individuals (N = 24). Mean kynurenine levels significantly increased 2 h post-LPS (*p* = 0.0008) and returned to baseline levels by 12 h post-LPS. This figure was created in GraphPad Prism v.9.1.1.

Inflammation is a risk factor for cardiometabolic disease. To understand a possible genetic relationship between kynurenine and CRP, an inflammatory biomarker associated with cardiometabolic disease risk, we conducted bi-directional Mendelian randomization analyses. Genetically determined kynurenine levels were associated with increased CRP (inverse-variance weighted average meta-analysis [IVWA] β = 0.21 (0.10), *p* = 0.04), but the reverse association was not significant (*p* = 0.71), suggesting that kynurenine may increase CRP levels. We also examined the effects of lipopolysaccharide (LPS) challenge (an acute inflammatory stimulus) on kynurenine levels in healthy human subjects (N = 24). In the Genetics of Evoked responses to Niacin and Endotoxemia **(**GENE) study, plasma kynurenine significantly increased by 25% (*p* = 0.0008) 2 h post-LPS challenge (Fig. 3), confirming that an inflammatory stimulus causes rapid increases in plasma kynurenine levels. This timepoint is concurrent with peak cytokine responses to LPS (e.g. TNFα, IL-6), but precedes an increase in CRP, which peaked 24 h post-LPS^16^, highlighting kynurenine as an early marker of acute inflammation.

### Kynurenine and body composition

Obesity is an inflammatory state, and is associated with elevated CRP levels^17^. We tested whether genetically determined central adiposity, measured by waist circumference is associated with kynurenine levels, and found a significant positive association (IVWA β = 0.04 (0.01), *p* = 0.0002). Similarly, in the ABO Study, measured waist circumference was positively correlated with plasma kynurenine levels (r = 0.279, *p* = 0.02). Collectively, these results suggest that central adiposity is associated with higher kynurenine levels.

### The kynurenine association with cardiometabolic diseases risk is specific to the *SH2B3* pathway

**Figure 4:**
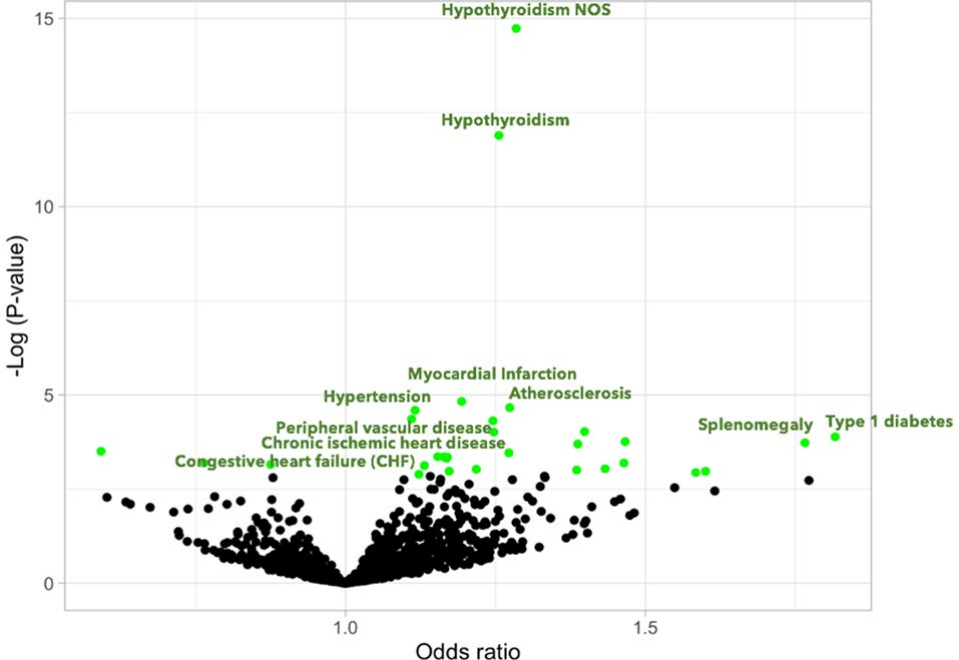
Volcano plot summarizing pheWAS associations for the *SH2B3* rs3184504 variant in the BioVU and eMERGE data sets. Each point indicates a phenotype association with T allele, from a logistic regression association analysis assuming an additive genetic model and are adjusted for age, gender and principal components. Odd-ratios greater than 1 indicated increased risk associated with increased kynurenine levels. Only some points are annotated for clarity, and points in green have FDR q < 0.05.

The individual SNPs associated with plasma kynurenine represent distinct mechanisms of kynurenine regulation and may have distinct patterns of disease associations. We conducted PheWAS for each of the six SNPs individually. The SNPs near the *IDO1/2* and *SLC7A5* genes, which directly contribute to either kynurenine synthesis or metabolism, had no significant associations with disease. In contrast, 30 phenotypes significantly associated with rs3184504 in *SH2B3* at FDR q < 0.05 (Supplementary Table 2). Among these associations were three distinct collections of diagnoses related to: hypothyroidism, hypertension and heart diseases including myocardial infarction, all of which were associated with higher kynurenine levels. The associations were similar in the Electronic Medical Records and Genetics (eMERGE)/BioVU (Fig. 4) and the UK Biobank data sets (Supplementary Fig. 2). In sum, the *SH2B3* rs3184504 variant associates with a range of diseases, and that previously reported epidemiological disease associations with plasma kynurenine may be driven by genetic mechanisms associated with *SH2B3*.

We performed phenome-wide association testing between the 6-SNP kynurenine polygenic risk score and 894 phenotypes in the BioVU and eMERGE datasets to explore whether genetically-predicted plasma kynurenine associated with clinical disease risk. We did not identify any significant associations (FDR q < 0.05). (Supplementary Fig. 3). Thus, plasma kynurenine is likely not a causal mediator of cardiometabolic diseases.

### The *SH2B3* rs3184504 variant may modulate disease though modulation of metabolic rate

We hypothesized that the mechanism linking *SH2B3* to disease may be through effects on energy metabolism. In the ABO study the rs3184504 risk variant was associated with significantly lower heart rate (β = −4.98, *p* = 0.0004, additive genetic model and β = −4.98, *p* = 0.017, recessive genetic model) and body temperature (β = −0.002, *p* = 0.0007, additive genetic model and β = −0.003, *p* = 0.004, recessive genetic model), suggestive of physiological differences. In the UK biobank **(**UKBB), we observed significant inverse relationship between the rs3184504 variant and whole body fat-free mass (β = −0.91, FDR *p* = 1.39E−23), and basal metabolic rate (β = −0.91, FDR *p* = 3.38E−21). However, this variant was not associated with body fat percentage (β = −0.05, FDR *p* = 0.89), suggesting a relationship with mitochondrial energy metabolism rather than a direct effect on adiposity.

**Figure 5:**
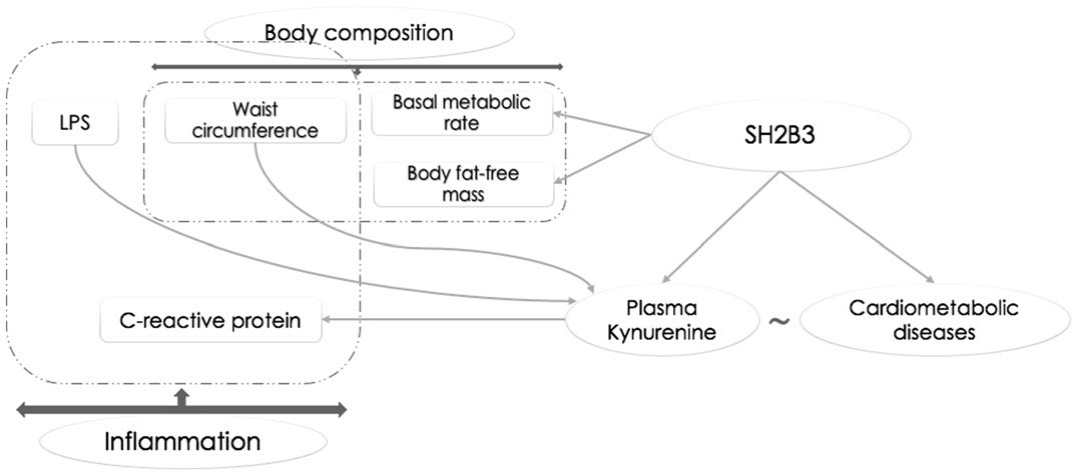
An overview of the study results.

Figure 5 summarizes the findings of the current study.

## Discussion

We leveraged genetic information to characterize the relationship between plasma levels of kynurenine and cardiometabolic diseases. Plasma kynurenine levels are determined by genetic variation associated with genes involved in tryptophan metabolism as well as genes without a clear previous link to kynurenine biosynthesis or catabolism. We confirmed a role for *SH2B3* regulation of kynurenine in a knock-out mouse model. Additional human studies (the GENE evoked endotoxemia study) also confirmed that kynurenine is induced by inflammatory stimuli and is likely up-stream of the inflammatory biomarker CRP, which is also associated, but not causally related, with multiple inflammatory diseases. A polygenic predictor that assimilated the genetics from all of the kynurenine-modulating mechanisms was not associated with clinical phenotypes, suggesting that plasma kynurenine itself does not cause disease. Indeed, the individual SNPs near genes with well-characterized roles in kynurenine metabolism (*IDO, SLC7A5*) were not associated with disease. However, we observed significant associations linking a kynurenine-modulating missense variant in *SH2B3* to a range of diseases including atherosclerosis, hypertension and hypothyroidism, as well as with white blood cell and platelet counts.

Genetic predictors of plasma kynurenine included some which impact established regulatory pathways; kynurenine is generated by tryptophan degradation by *IDO*, the first and rate-limiting enzyme in tryptophan catabolism, whose activity is stimulated by inflammation^3,10^. *SLC5A7* encodes a protein involved in tryptophan transport across the cell membrane^11^. *IDO* and *SLC5A7* are thought to predominately regulate kynurenine production. However, neither *IDO* or *SLC5A7* individually, or polygenic risk score analyses, showed genetic associations with cardiometabolic disorders or other disease. These data suggest that plasma kynurenine levels per se do not have a causal relationship to these diseases.

The *SH2B3* rs3184504 variant was associated with many diseases, including cardiometabolic and thyroid disease. This variant is a nonsynonymous SNP located in exon 3 of the *SH2B3* gene which leads to a R262W amino acid change and has been shown to be involved in controlling immune responses. *SH2B3* (*LNK*) is important in hematopoiesis, regulates the expansion of dendritic cells in lymph nodes, acts as a negative regulator of cytokine signaling, and has been associated with increased susceptibility to aortic dissection^18–20^. It is thought that the rs3184504 variant in *SH2B3* approximates a loss of function^21^. Thus, to confirm the relationship between *SH2B3* and kynurenine, we measured kynurenine levels in *SH2B3*^*−*/*−*^mice, and observed significant plasma and tissue-specific changes in kynurenine.

Up to now, no mechanistic relationship between *SH2B3* and kynurenine metabolism had been reported. *SH2B3* may regulate kynurenine levels indirectly through modulation of inflammatory signaling, and subsequent activation of *IDO*^22^. However, our data supported an association between rs3184504 and kynurenine in healthy individuals, without concurrent inflammation, suggesting *SH2B3* may directly modulate the kynurenine pathway.

The kynurenine pathway controls production of NAD + , with important consequences for energy metabolism, and a known relationship to acute kidney injury^23^. Our data support a link between the SH2B3-kynurenine axis and energy homeostasis. We found that the *SH2B3* rs3184504 variant associates with lower whole body fat-free mass and basal metabolic rate, in addition to lower body temperature and heart rate. There may be an important distinction between kynurenine that is elevated due to increased synthesis from tryptophan (controlled by *IDO* and *SLC7A5*), versus kynurenine that is elevated due to reduced breakdown of kynurenine or altered downstream pathway flux (potentially controlled by *SH2B3*). This hypothesis remains to be further interrogated, and there may be other mechanisms linking *SH2B3* to kynurenine, which remain to be explored.

Epidemiological studies have shown that higher plasma kynurenine levels associate with increased inflammation and CVD prevalence in patients with end-stage renal disease. Individuals with higher kynurenic acid levels were more likely to have higher risk of coronary artery disease mortality and myocardial infarction^24^. Furthermore, kynurenine has been associated with the risk of all-cause mortality, particularly death from CVD^25^. We found that plasma kynurenine was acutely regulated during evoked endotoxemia, suggestive of a role in acute inflammatory responses. Kynurenine has recently been reported to be upregulated during COVID-19 infection, further highlighting a potential role for kynurenine as a biomarker of inflammatory activation^26^.

Waist circumference, a marker of central obesity, was positively associated with kynurenine. We also observed an inverse association between the rs3184504 variant and whole body fat-free mass and basal metabolic rate. PheWAS analyses revealed that the rs3184504 variant was directly associated with some obesity associated co-morbidities including hypertension and heart-related diseases, but not some other obesity-related comorbidities including obstructive sleep apnea and type 2 diabetes. Consistent with these findings, previous research demonstrated that *SH2B3*-related genetic alterations contribute to the development of hypertension and hematological disorders^27,28^. Consistently, the *SH2B3* rs3184504 variant is associated with increased risk of CAD^29^, increased platelet counts and leukocytosis^30^, diastolic blood pressure^31^, atherosclerosis and thrombosis^21^. Thus, these data suggest that this *SH2B3*-related mechanism, which regulates both disease risk and kynurenine levels, may account for the observed associations between plasma kynurenine and the risk of cardiometabolic diseases. Traditional prospective studies seeking to link a metabolomic biomarker to a disease are limited based on sample sizes, follow-up times and do not provide insights into the molecular mechanisms account for candidate associations. We circumvented these limitations by using an approach which associated a putative biomarker with clinical traits based on their shared genetic structures. Thus, we were able probe for kynurenine associations in large, deeply phenotyped populations. Along with other strengths (increasing sample sizes and the number of diseases), this approach can also identify molecular mechanisms underlying an association. Another strength of our study is that we replicated findings in multiple populations (BioVU, eMERGE and the UKBB).

Our study had considerable strengths, but also some limitations. Using EHR billing codes rather than a systematic ascertainment of a diagnosis for classifying PheWAS phenotypes in BioVU and eMERGE databases might lead to both false-positive and false-negative associations. To address this, we replicated the study in other databases including UKBB and published data from large disease GWAS. Negative associations, such as the absence of an association between the kynurenine polygenic score (PRS) and disease, leave open the possibility of a false negative due to lack of power. However, we obtained very strong associations for the SNP in *SH2B3* in the same datasets, indicating that the kynurenine genetic instruments should have been suitably powered. While experimental evidence is provided for showing kynurenine increases associated with acute inflammatory stimuli, additional studies are needed to confirm sustained elevations associated with inflammatory states. Furthermore, tissues in the heart, lungs and cardiopulmonary vasculature were not available for analyses and differences in levels in KO mice could not be assessed.

In conclusion: in this virtual biomarker study, we explored the association between kynurenine genetic predictors and clinical diagnoses derived from large datasets. Our findings suggest diverse molecular mechanisms regulate plasma kynurenine. The *SH2B3-*rs3184504 variant associates with both plasma kynurenine and diseases; our data suggest this is independent of kynurenine production. Plasma kynurenine, upregulated during inflammation, is upstream of the inflammatory biomarker CRP. The *SH2B3* rs3184504 variant, which regulates kynurenine levels, associates with increased cardiometabolic disorders, potentially in a kynurenine-independent manner. In sum, although targeting plasma kynurenine directly is unlikely to be effective in disease treatment, interrogation of the *SH2B3* pathways during inflammation may identify novel causal disease mechanisms.

## Materials and methods

We identified SNPs associated with plasma kynurenine from published data^32^, and tested their associations with disease phenotypes. Identified associations were validated and probed using animal models and in independent populations.

### Kynurenine GWAS summary statistics

SNPs associated with plasma kynurenine were identified from a GWAS meta-analysis of the KORA-TwinsUK studies^32^. Analyses were based on 7824 adult individuals of European descent^33,34^. Summary statistics were obtained from the Metabolomics GWAS server (http://metabolomics.helmholtz-muenchen.de/gwas/)^32^. An independent (r^2^ < 0.05 within 1000 kilobases) set of SNPs significantly associated (*p* < 5 × 10^−8^) with circulating kynurenine levels were selected from the KORA-TwinsUK meta-analysis GWAS summary statistics.

### Electronic health record-linked genetic datasets

#### BioVU

BioVU is Vanderbilt University Medical Center’s DNA biobank, which is linked to de-identified EHR phenotype data^35^. A subset of BioVU (n = 62,303) participants of European Ancestry have SNP genotype data acquired on the Illumina MEGA^EX^ platform. Quality control steps for the BioVU population have been previously described^36^. Genotypes were imputed with IMPUTE4^37^, version 2.3.0 (University of Oxford), using the 10/2014 release of the 1000 Genomes cosmopolitan reference haplotypes and variants imputation quality scores less than 0.3 were excluded. One participant from each related pair (pi-hat > 0.2) was randomly excluded. Analyses were restricted to subjects of European ancestry, defined by principal components analyses in conjunction with HapMap reference populations. Quality control analyses used PLINK v1.9^38^. The use of BioVU and other de-identified data presented in these analyses was approved by the VUMC Institutional Review Board (IRB), in accordance with the informed consent guidelines.

### eMERGE

The eMERGE Phase I, II and III Network, a consortium of medical centers using EHRs as a tool for genomic research, included^39^ participants (n = 32,324) who were born prior to 1990 and were recruited from Geisinger Health System, Marshfield Clinic, Northwestern University, Mayo Clinic, and Kaiser Permanente/University of Washington. Consent was collected based on each site’s IRB protocols. eMERGE data were genotyped on multiple SNP arrays. QC procedures and imputation protocols for these data were conducted based on the established protocols developed by the eMERGE Genomics Working Group^41^. Use of de-identified eMERGE data was approved by the IRB at each site^39^, in accordance with the site-specific informed consent guidelines.

#### The UKBB study

UKBB is a British population-based self-reported study which is composed of approximately 0.5 million participants aged 37–73 at recruitment^42^. GWAS summary statistics for 2173 UKBB phenotypes^43^ were downloaded from the study by Bycroft et al.^44^. PheWAS results for individual kynurenine-associated SNPs were obtained from http://geneatlas.roslin.ed.ac.uk/^43^.

### Disease genome-wide association study datasets

Summary statistics for CRP were obtained from a GWAS meta-analysis of 204,402 European individuals^45^. Additional summary statistics were downloaded from published GWAS of waist circumference^20^.

### Phenome-wide Association Analysis (PheWAS)

Single SNP and multi SNP PheWAS analyses were conducted by testing associations with either individual SNPs (single SNP) associated with kynurenine or all kynurenine SNPs (a PRS comprising multi SNPs) PheWAS phenotypes. Analyses used the R PheWAS package^46^.

PheWAS were performed in BioVU and eMERGE using clinical phecode phenotypes (https://phewas.mc.vanderbilt.edu/) based on ICD-9-CM and ICD-10 diagnosis codes^47,48^. Individuals with two or more instances of a PheWAS diagnosis existing in their medical documents were considered as cases^49^. Clinical phenotypes with ≥ 300 cases were included and those affecting a single sex (like uterine prolapse and prostate cancer) were excluded. After exclusions, there were 894 phenotypes. Controls were individuals without any closely related PheWAS codes, and were matched to the age (BioVU) or decade of birth (eMERGE) ranges among the cases.

### eQTL analysis

eQTL data for selected SNPs were obtained from the Genotype Tissue Expression (GTEx) portal, https://gtexportal.org.

### Animal models

#### SH2B3^−/−^ mouse

Plasma and tissues (white adipose tissue, brain, liver, spleen) were obtained from 14–15 week-old C57BL/6 J mice (wild type [WT, N = 9 plasma, N = 8 tissues)]) and from Lnk^−/−^ (Sh2b3 knock out [KO], N = 5), as previously described^50^. Samples were frozen immediately following collection, and stored at − 80° C prior to analysis. Tissue samples were homogenized (Tissue Lyser LT) and stored at − 80° C prior to metabolite measurement. Mice were housed and taken care of in accordance with the Guide for the Care and Use of Laboratory Animals, US Department of Health and Human Services. All animal procedures were approved by the Vanderbilt Institutional Animal Care and Use Committee.

### Enzyme-linked immunosorbent assay (ELISA) of kynurenine

Kynurenine in mouse plasma and tissue extracts was analyzed by ELISA (Cod. LS-F56401, *LifeSpan BioScience Inc*., Seattle, WA, USA), according to manufacturer’s instructions. All samples were run in duplicate, with an even distribution of samples from KO and WT animals across the three plates used. The intra-assay coefficient of variance was 9.8%. Due to relatively high inter-assay variability, likely attributable to differences by lot in the three ELISA kits used, we analyzed data as fold difference between WT and KO mice for each plate, rather than absolute values.

### Clinical studies

#### GENE study

Healthy volunteers (294 non-pregnant/lactating women and men, age 18–45, BMI 18–30 kg/m^2^) were recruited to an evoked endotoxemia study (LPS, 1 ng/kg) at the University of Pennsylvania, as previously described^16^. Plasma metabolites, including kynurenine, were measured in a subset of individuals (n = 24) at baseline, and two hours post LPS-challenge, by mass spectrometry in positive and negative ion modes using well-established protocols^51,52^.

#### ABO study

The ABO Study recruited healthy volunteers (non-pregnant/lactating women and men, age 18–50) to a single study visit at the University of Pennsylvania from 2012 to 2014, as described^53^. Plasma metabolomics profiling, including measurement of kynurenine, was carried out at Metabolon (Metabolon Inc, Morrisville, NC; global metabolomics platform). Genotyping was performed using the Exome chip (Illumina, CoreExome, N > 540,000 variants, including rs3184504) at the Center for Applied Genomics at the Children's Hospital of Philadelphia. We analyzed data for a subset of individuals with overlapping metabolite and genetic data (N = 66).

The GENE and ABO studies were approved by the IRB of the University of Pennsylvania and Vanderbilt University. All participants provided written informed consent.

### Statistical analyses

#### Genotyped populations (UKBB, BioVU, eMERGE and other large disease GWAS)

##### SingleSNP

PheWAS analyses for each kynurenine-associated SNP were performed in the BioVU and eMERGE populations. Associations were tested assuming an additive genetic model and used multivariable logistic regression models adjusting for 5 PCs, sex and either birth decade (eMERGE) or age (BioVU). BioVU and eMERGE data were combined using meta-analyses encoded by the METAL package (default settings were used)^54^.

##### MultiSNP

A PRS for plasma kynurenine levels was computed for each individual in the BioVU and eMERGE populations by summing their (Allele dosage x change in kynurenine levels per allele) for each kynurenine-associated SNPs. Association testing between the PRS and each phenotype was then tested and combined, as described above. Odds-ratios (ORs) represent the risk of disease per standard deviation (SD) increase in the PRS.

For UKBB phenotypes, and additional phenotypes based on GWAS summary statistics, mutliSNP association tests were conducted. Associations were tested using the IVWA, MR-Egger and Weighted Median methods, as implemented in the Mendelian Randomization R package^55^. Heterogeneity *p* values are based on the Cochran’s Q statistic, and a low *p* value may indicate horizontal pleiotropy. Association estimates represent the change in the log odds-ratio per standard deviation change in plasma kynurenine.

##### Multiple testing corrections

We applied a strict Benjamini–Hochberg (B–H) false discovery rate (FDR)^56^ to adjust for multiple testing, and associations with a q-value < 0.05 were considered significant^40^. All analyses were performed in accordance with relevant guidelines and regulations. The study was carried out in compliance with the ARRIVE guidelines.

## Supplementary Information

Supplementary Information 1.

Supplementary Information 2.

Supplementary Information 3.

## Data Availability

eMERGE data are available through dbGaP (phs000360.v3.p1). Upon acceptance, the complete findings from the PheWAS analyses will be made available thought a publicly-available website.

## Abbreviations

B–H: Benjamini–Hochberg
CRP: C-reactive protein
CVD: Cardiovascular diseases
ELISA: Enzyme-linked immunosorbent assay
eMERGE: Electronic medical records and genetics consortium
eQTL: Expression quantitative trait loci
FDR: False discovery rate
GENE: Genetics of evoked responses to niacin and endotoxemia
GTEx: Genotype tissue expression
3HAA: 3-Hydroxy anthranilic acid
HK: Hydroxykynurenine
IDO: Indoleamine 2,3-dioxygenase
IRB: Institutional Review Board
IVWA: Inverse-variance weighted average
KORA: Cooperative Health Research in the Region of Augsburg
KA: Kynurenic acid
LD: Linkage disequilibrium
LPS: Lipopolysaccharide
NA: Nicotinic acid
NAM: Nicotinamide
OR: Odds-ratio
PheWAS: Phenome-wide association analysis
PRS: Polygenic score
QA: Quinolinic acid
SD: Standard deviation
SNP: Single nucleotide polymorphism
