# Supplementary material for: The genetic architecture of plasma kynurenine includes cardiometabolic disease mechanisms associated with the SH2B3 gene
Source: Sci Rep. 2021 Aug 2;11:15652. doi: 10.1038/s41598-021-95154-9 (PMC8329184; doi:10.1038/s41598-021-95154-9)
Supplement: Supplementary file 1 — Supplementary Information 1. [file 41598_2021_95154_MOESM1_ESM.docx]

**Title:** **The Genetic Architecture of Plasma Kynurenine includes Cardiometabolic Disease Mechanisms Associated with the *SH2B3* Gene**

**Authors:** Minoo Bagheri,^1^ Chuan Wang,^1^ Mingjian Shi^2^, Ali Manouchehri,^1,3^ Katherine T. Murray,^1,3^ Matthew B. Murphy,^3^ Christian M. Shaffer,^2^ Kritika Singh,^4^ Lea K. Davis,^4^ Gail P. Jarvik,^5^ Ian B. Stanaway,^6^ Scott Hebbring,^7^ Muredach P. Reilly,^8^ Robert E. Gerszten,^9^ Thomas J. Wang,^10^ Jonathan D. Mosley,*^2,3^ Jane F. Ferguson*^1^

*Authors contributed equally

**Affiliations:**^1^ Division of Cardiovascular Medicine, Department of Medicine, Vanderbilt University Medical Center, Nashville TN. [bagheriminoo@yahoo.com](mailto:bagheriminoo@yahoo.com), [chuan.wang@vumc.org](mailto:chuan.wang@vumc.org), [ali.m.manouchehri@vumc.org](mailto:ali.m.manouchehri@vumc.org), [jane.f.ferguson@vumc.org](mailto:jane.f.ferguson@vumc.org)

^2^ Department of Biomedical Informatics, Vanderbilt University Medical Center, Nashville TN. [mingjian.shi.1@vumc.org](mailto:mingjian.shi.1@vumc.org), [christian.m.shaffer@vumc.org](mailto:christian.m.shaffer@vumc.org), [jonathan.d.mosley@vumc.org](mailto:jonathan.d.mosley@vumc.org)

^3^ Division of Clinical Pharmacology, Department of Medicine, Vanderbilt University Medical Center, Nashville TN. [kathy.murray@vumc.org](mailto:kathy.murray@vumc.org), [matthew.b.murphy@vanderbilt.edu](mailto:matthew.b.murphy@vanderbilt.edu)

^4^ Division of Genetic Medicine, Department of Medicine, Vanderbilt University Medical Center, Nashville, TN, USA. [kritika.singh@vanderbilt.edu](mailto:kritika.singh@vanderbilt.edu), [lea.k.davis@vumc.org](mailto:lea.k.davis@vumc.org)

^5^ Departments of Medicine (Medical Genetics) and Genome Sciences, University of Washington, Seattle, WA, USA. [gjarvik@medicine.washington.edu](mailto:gjarvik@medicine.washington.edu)

^6^ Harborview Medical Center Kidney Research Institute, Division of Nephrology, School of Medicine, University of Washington, Seattle, WA. [bard@uw.edu](mailto:bard@uw.edu)

^7^ Center for Precision Medicine Research, Marshfield Clinic Research Institute, Marshfield, Wisconsin USA. [hebbring.scott@marshfieldresearch.org](mailto:hebbring.scott@marshfieldresearch.org)

^8^ Irving Institute for Clinical and Translational Research and Division of Cardiology, Columbia University Medical Center, New York, NY. [mpr2144@cumc.columbia.edu](mailto:mpr2144@cumc.columbia.edu)

^9^ Beth Israel Deaconess Medical Center, Division of Cardiovascular Medicine, Boston, Massachusetts. [rgerszte@bidmc.harvard.edu](mailto:rgerszte@bidmc.harvard.edu)

^10^ Department of Internal Medicine, University of Texas Southwestern Medical Center. [thomas.wang@utsouthwestern.edu](mailto:thomas.wang@utsouthwestern.edu)

**Correspondence:**

Jane F. Ferguson, PhD

Vanderbilt University Medical Center

2220 Pierce Ave, PRB 354B

Nashville, Tennessee 37232

Tel: 615-875-9896

Email: [jane.f.ferguson@vumc.org](mailto:jane.f.ferguson@vumc.org)

| 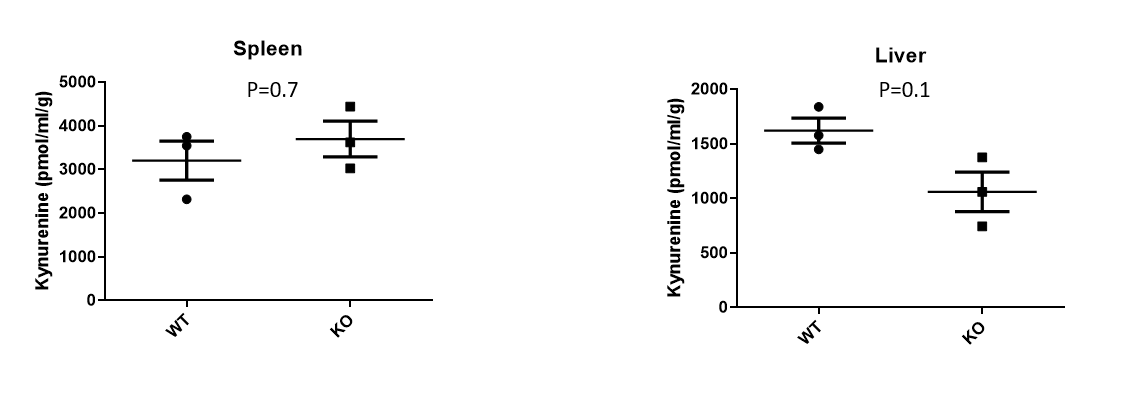 |
| --- |
| Supplementary Fig. 1. Kynurenine levels in selected tissues in wild type (WT, N=3) and *SH2B3* knockout (KO, N=3) mice. Kynurenine levels were measured by ELISA assay. Values are expressed as pmol/ml, normalized to tissue weight in grams. Differences between WT and KO animals were analyzed by unpaired t test. |

|  | 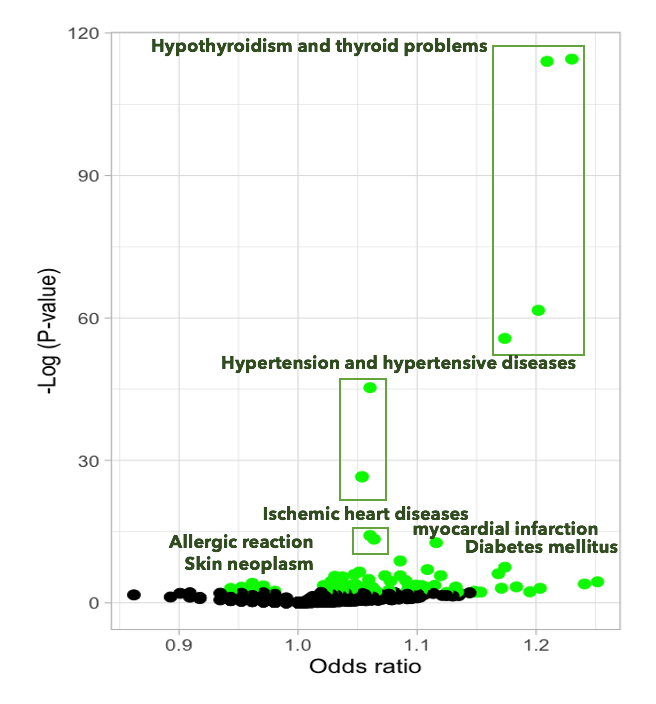 |
| --- | --- |
| Supplementary Fig. 2- Volcano plot summarizing pheWAS associations for the *SH2B3* rs3184504 variant in the UKBB data set. Each point indicates a phenotype association with T allele, from a logistic regression association analysis assuming an additive genetic model and are adjusted for age, gender and principal components. Odd-ratios greater than 1 indicated increased risk associated with increased kynurenine levels. Only some points are annotated for clarity, and points in green have FDR q<0.05. | |

| 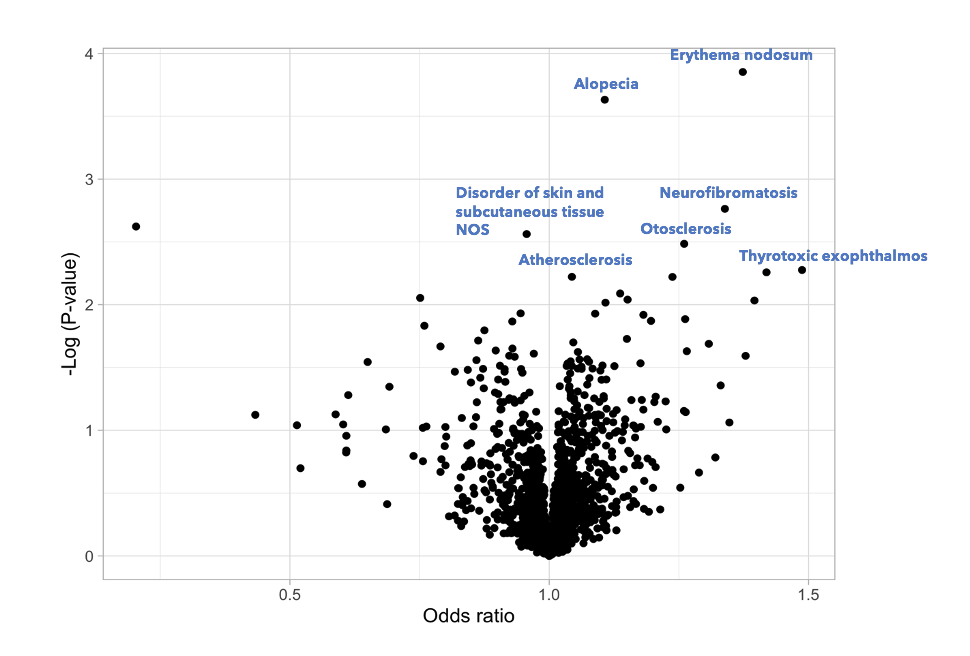 |
| --- |
| Supplementary Fig. 3- Volcano plot summarizing pheWAS analyses for a polygenic genetic predictor of plasma Kynurenine in the meta-analysis of BioVU and eMERGE studies. Each point represents the results of an associations test with a clinical phenotype. Results are from a logistic regression model. Only some points are annotated for clarity. |
