# Supplementary material for: The genetic architecture of plasma kynurenine includes cardiometabolic disease mechanisms associated with the SH2B3 gene
Source: Sci Rep. 2021 Aug 2;11:15652. doi: 10.1038/s41598-021-95154-9 (PMC8329184; doi:10.1038/s41598-021-95154-9)
Supplement: Supplementary file 2 — Supplementary Information 2. [file 41598_2021_95154_MOESM2_ESM.docx]

| **Supplementary Table 1-** Top GWAS loci associated with downstream metabolites of the Kynurenine pathways^*^ | | | | | | | | |
| --- | --- | --- | --- | --- | --- | --- | --- | --- |
| **Metabolite** | **SNP** | **Chromosome** | **Position** | **Reference Allele** | **Alternative Allele** | **BETA** | **SE** | **P-value** |
| Quinolinic acid | rs12461592 | 18 | 12461592 | T | A | -0.52 | 0.31 | 2.53E-08 |
| Quinolinic acid | rs11686981 | 2 | 211097828 | G | C | 0.41 | 0.07 | 3.40E-08 |
| Quinolinic acid | rs13385593 | 2 | 217726821 | G | A | 0.65 | 0.12 | 3.17E-08 |
| Quinolinic acid | rs17773128 | 2 | 211122296 | C | T | 0.40 | 0.07 | 4.55E-08 |
| Quinolinic acid | rs2164920 | 3 | 72603331 | G | A | -0.30 | 0.05 | 4.73E-08 |
| Quinolinic acid | rs367923752 | 9 | 102016 | C | G | -0.41 | 0.31 | 9.73E-09 |
| Quinolinic acid | rs6752042 | 2 | 211094909 | T | A | 0.41 | 0.07 | 3.17E-08 |
| Nicotinamide | rs11608136 | 11 | 85912564 | T | G | 0.22 | 0.05 | 4.26E-08 |
| Nicotinamide | rs2084078 | 11 | 85920988 | G | A | 0.22 | 0.05 | 3.56E-08 |
| Nicotinamide | rs4944546 | 11 | 85916441 | C | A | 0.22 | 0.05 | 2.39E-08 |

^*^ Data was obtained from Rhee et al.^1^ and Long et al.^2^ studies

References:

1. Rhee EP, Ho JE, Chen M-H, Shen D, Cheng S, Larson MG, Ghorbani A, Shi X, Helenius IT, O’Donnell CJ, Souza AL, Deik A, Pierce KA, Bullock K, Walford GA, Vasan RS, Florez JC, Clish C, Yeh J-RJ, Wang TJ, Gerszten RE. A genome-wide association study of the human metabolome in a community-based cohort. *Cell Metab*. 2013;18:130–143.

2. Long T, Hicks M, Yu H-C, Biggs WH, Kirkness EF, Menni C, Zierer J, Small KS, Mangino M, Messier H, Brewerton S, Turpaz Y, Perkins BA, Evans AM, Miller LAD, Guo L, Caskey CT, Schork NJ, Garner C, Spector TD, Venter JC, Telenti A. Whole-genome sequencing identifies common-to-rare variants associated with human blood metabolites. *Nat Genet*. 2017;49:568–578.
